# Malaria-Antigene in der Ära der mRNA-Impfstoffe: Mögliche Rettungen vor einer der relevantesten und gefährlichsten Kinderkrankheiten des Globalen Südens

**DOI:** 10.1007/s00112-022-01554-0

**Published:** 2022-07-14

**Authors:** Yannick Borkens

**Affiliations:** College of Public Health, Medical and Veterinary Science, James Cook University, 1 James Cook Drive, 4811 Townsville, Queensland Australien

**Keywords:** Malaria, mRNA-Impfstoffe, Parasitische Infektionskrankheiten, Plasmodium, Tropenmedizin, Malaria, mRNA vaccines, Parasitic infectious diseases, Plasmodium, Tropical medicine

## Abstract

Bereits in den frühen 1990er-Jahren wurde erstmals eine durch einen mRNA-Impfstoff ausgelöste Immunantwort beschrieben. Seitdem wurden mRNA-Impfstoffe für eine mögliche Prophylaxe erforscht und diskutiert. Doch erst mit der COVID-19-Pandemie erlebten diese Impfstoffe einen wahren Boom. Die ersten mRNA-Impfstoffe wurden gegen SARS-CoV‑2 zugelassen und zeigten große Erfolge. Es ist daher nicht verwunderlich, dass sich die Hersteller auch auf andere Krankheiten und Pathogene konzentrieren. Neben viralen Krankheiten wie Influenza oder Aids steht Malaria weit oben auf dieser Liste. Viele Pharmaunternehmen (u. a. die deutschen Unternehmen BioNTech und CureVac) haben bereits bestätigt, an mRNA-Impfstoffen gegen Malaria zu forschen. Dabei ist die Entwicklung eines funktionierenden Impfstoffes gegen Malaria kein leichtes Unterfangen. Seit den 1960ern wird an möglichen Impfstoffen geforscht. Die Ergebnisse sind dabei eher ernüchternd. Erst 2015 erhielt der Impfstoff RTS,S/AS01 eine positive Bewertung der Europäischen Arzneimittel-Agentur. Seitdem wird der Impfstoff in Afrika getestet.

## Abstract (en)

Even in the early 1990s, an immune response triggered by an mRNA vaccine was described for the first time. Since then, mRNA vaccines have been researched and discussed for possible prophylaxis; however, it was not until the COVID-19 pandemic that these vaccines experienced a real boom. The first mRNA vaccines were licensed against SARS-CoV‑2 and showed great success. It is therefore not surprising that manufacturers are also focusing on other diseases and pathogens. Besides viral diseases, such as influenza and AIDS, malaria is high on this list. Many pharmaceutical companies (including the German companies BioNTech and CureVac) have already confirmed that they are researching mRNA vaccines against malaria. Yet developing a working vaccine against malaria is no easy feat. Research on possible vaccines has been going on since the 1960s. The results have been rather sobering. It was not until 2015 that the vaccine RTS,S/AS01 received a positive evaluation from the European Medicines Agency. Since then, the vaccine has been tested in Africa.

## Entwicklung der ersten mRNA-Impfstoffe

1961 wurde mRNA zum ersten Mal als Informationsträger für die Biosynthese entdeckt. Dies wurde von Brenner et al. beschrieben [1]. Diese Entdeckung gilt als Grundstein für eine Reihe weiterer Entdeckungen und Entwicklungen. Dazu gehört auch die Entwicklung von Arzneimitteln auf der Grundlage von mRNA. Das erste RNA-basierte Medikament, Patisiran, wurde 2018 von der EMA (Europa) und der FDA (USA) zugelassen. Patisiran, das unter dem Namen Onpattro® vermarktet wird, wurde von dem in Cambridge ansässigen Unternehmen Alnylam® Pharmaceuticals für die Behandlung von ATTR-Amyloidose, einer seltenen Form der Amyloidose, entwickelt [2, 60]. Der Wirkmechanismus des Medikaments basiert auf dem „RNA silencing“, bei dem Gene gezielt ausgeschaltet werden.

**Figure 1:**
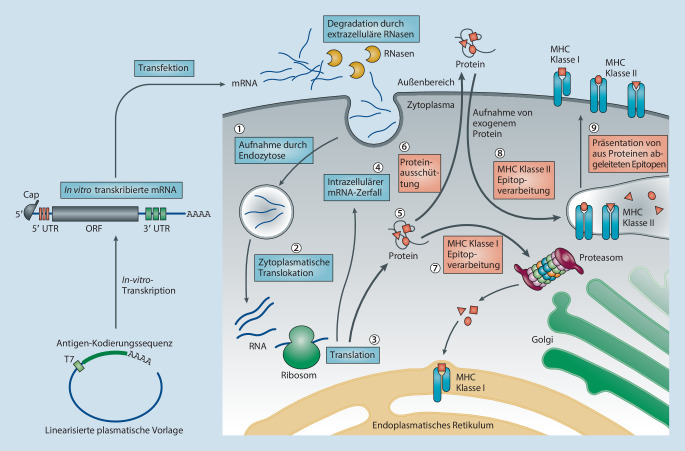

Die ersten RNA-basierten Impfstoffe wurden in den Jahren 1993 und 1994 beschrieben. In diesen Jahren wurde bereits eine zelluläre Immunantwort induziert [3, 61]. Im Jahr 1995 konnte mit der RNA-Impfung auch eine humorale Immunantwort induziert werden [3, 4]. Seit Anfang der 2000er-Jahre wird an der RNA-Impfung für Menschen geforscht, zu Beginn mit mäßigem Erfolg [62]. Erst 2017 wurden RNA-Impfstoffe von der WHO als neue Impfstoffklasse zugelassen [5]. Die COVID-19-Pandemie von 2019 führte zum endgültigen Durchbruch der mRNA-basierten Impfstoffe [6]. Bereits 2020 wurden die ersten mRNA-Impfstoffe gegen COVID-19 zugelassen (BNT162b2 von Pfizer/BioNTech und mRNA-1273 von Moderna). Beide Impfstoffe erzielten sehr gute Ergebnisse [7, 8]. Im Gegensatz zu inaktivierten Impfstoffen wird bei der mRNA-Impfung lediglich eine mRNA, die für ein spezifisches Impfantigen kodiert, in die Zelle eingebracht. Die Zelle beginnt, das Antigen auf der Basis der eingebrachten mRNA zu produzieren, und präsentiert es den immunreaktiven Zellen. Daraufhin reagiert das Immunsystem auf das Antigen und ist somit in der Lage, das Antigen im natürlichen Erreger zu erkennen und den Wirt zu schützen. Abb. 1 veranschaulicht den Wirkmechanismus schematisch. Als Antigene werden v. a. Oberflächenproteine verwendet. Bereits die Entwicklung von COVID-19-Impfstoffen sowie der generelle Durchbruch von mRNA-Impfstoffen werden von einigen als nobelpreiswürdig bezeichnet [9].

## Historischer Hintergrund der Malaria-Impfstoffe

**Figure 2:**
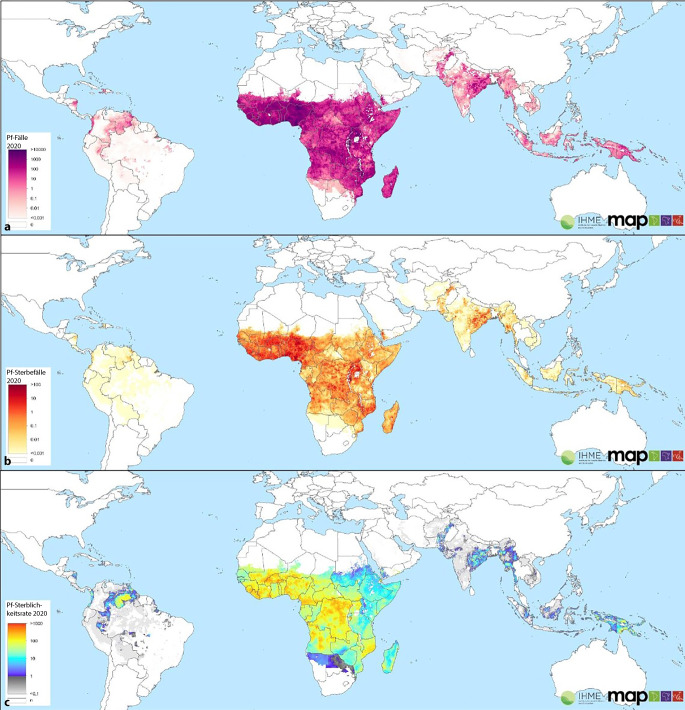

Malaria ist eine tropentypische Krankheit und weltweit nicht nur eine der bedeutendsten, sondern auch eine der tödlichsten Infektionskrankheiten [10]. Ungefähr 40 % der Weltbevölkerung leben in Malaria-Endemiegebieten. Pro Jahr erkranken ca. 200 Mio. Menschen. Von diesen sterben ungefähr 600.000. Etwa drei Viertel von ihnen sind Kinder unter 5 Jahren. Darüber hinaus gilt Malaria als Todesursache von prominenten Persönlichkeiten innerhalb der Menschheitsgeschichte.^1^ Zu diesen gehören z. B. Tutanchamun, der Westgotenkönig Alaric und Papst Innozenz VIII. [63]. Obwohl es sich bei diesen Punkten nicht um Fakten handelt und sie in der wissenschaftlichen Gemeinschaft sicherlich umstritten sind, besteht kein Zweifel daran, dass Malaria eines der wichtigsten Probleme ist, mit denen die globalen Gesundheitssysteme konfrontiert sind. Diese Bedeutung wird durch die Tatsache verstärkt, dass Malaria v. a. arme Länder im Globalen Süden (Afrika, Asien und Südamerika) betrifft [11, 12, 64]. Die WHO gibt an, dass 67 % der Todesfälle unter 5 Jahren auftreten. Abb. 2 zeigt die Fälle, Todesfälle und Sterblichkeitsrate von *Plasmodium falciparum* im Jahr 2020. Die Abbildung wurde dem Malaria Atlas Project entnommen, einem Collaborating Centre in Geospital Disease Modelling der WHO. Das Projekt wurde 2005 ins Leben gerufen, um eine Nische für die Malariabekämpfung auf globaler Ebene zu füllen [13, 14, 65]. Viele verschiedene Unternehmen sind aktuell an funktionierenden Malariaimpfstoffe interessiert. Zu diesen gehören u. a. die deutschen Unternehmen BioNTech aus Mainz und CureVac aus Tübingen.^2^^,^^3^ Aber auch Länder wie Deutschland, die Vereinigten Staaten und andere ziehen eine effektive Malariabekämpfung zunehmend in Betracht.^4^^,^^5^

Die Entwicklung eines wirksamen Malaria-Impfstoffs ist jedoch keineswegs neu. Nachdem die Malaria in den 1960er-Jahren (durch gezielte Public-Health-Maßnahmen und Programme während des Zweiten Weltkriegs) als besiegt galt, stiegen die Fälle Ende der 1960er-Jahre wieder stark an. Dies war v. a. auf die Resistenz gegen DDT und Chloroquin zurückzuführen, die *Plasmodium* im Laufe der Jahre entwickelte. Zu Beginn dieses Zeitraums wurde klar, dass nur eine Impfung die Krankheit stoppen konnte [15]. Dass Impfstoffe das Potenzial haben, gefährliche Krankheiten zu regulieren oder sogar vollständig auszurotten, zeigt die Pockenimpfkampagne. Die Pocken wurden 1980 von der WHO dank einer beispiellosen weltweiten Zusammenarbeit für ausgerottet erklärt [16]. Da im Falle von Malaria jedoch auch Primaten als Wirte infrage kommen, ist eine Ausrottung über Impfungen recht unwahrscheinlich.

**Figure 3:**
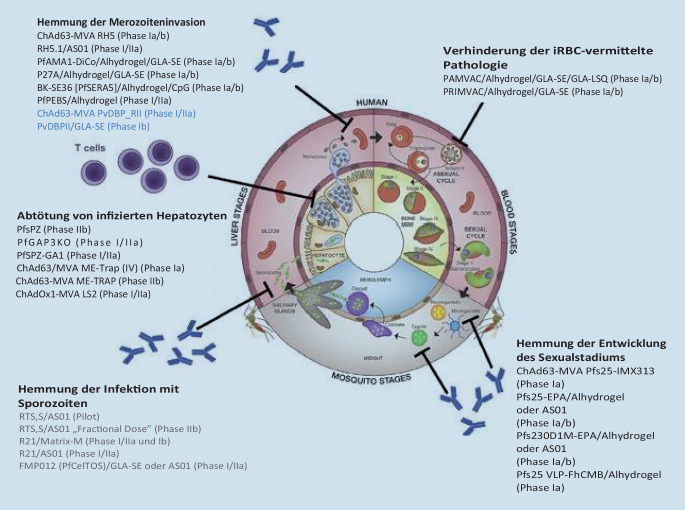

Heute gibt es verschiedene Impfstoffplattformen mit unterschiedlichen Wirkmechanismen. Diese unterscheiden sich durch ihren Wirkort und ihre Wirkungsweise. Es gibt 3 verschiedene Impfstoffklassen: „pre-erythrocytic vaccines“, „blood-stage vaccines“ und „transmission blocking vaccines“. Während Pre-Erythrocytic vaccines und Blood-Stage vaccines in erster Linie eine Immunantwort auslösen sollen, sollen Transmission blocking vaccines die Übertragung auf den Menschen und damit die Ausbreitung der Krankheit verhindern [17]. Abb. 3 zeigt eine schematische Darstellung der verschiedenen Impfstoffplattformen gegen Malaria. Die Malariaerreger der Gattung *Plasmodium* werden von Stechmücken der Gattung *Anopheles* übertragen [66, 67]. Die Gattung *Anopheles* umfasst etwa 420 Arten, von denen etwa 40 Arten Malaria übertragen. *Anopheles*-Mücken kommen weltweit in den Tropen und Subtropen vor (mit Ausnahme der meisten Inseln in Ozeanien wie Neuseeland, Fidschi oder Neukaledonien). Darüber hinaus gibt es auch Berichte über *Anopheles* in gemäßigten Regionen. Neuere Aufzeichnungen berichten auch über Arten in kälteren Ländern wie Finnland [18–20, 68]. Aus diesem Grund spielt die Vektorkontrolle eine wichtige Rolle bei der Malariabekämpfung [21, 69]. Transmission blocking vaccines sind Teil dieser Vektorkontrollmaßnahmen. Sie wirken auf die Gametozyten und zielen auf spezifische Proteine ab, die eine wichtige Rolle bei der Gametozytenentwicklung spielen. Eines dieser Proteine ist Pfs48/45, das für die Entwicklung männlicher Mikrogametozyten wichtig ist [22].

### Serum *Plasmodium falciparum* Version 66

Der erste echte Impfstoff gegen Malaria gehört jedoch zu den Blutstadienimpfstoffen, d. h. zu den Impfstoffen, die sich auf das Blutstadium des *Plasmodium*-Lebenszyklus konzentrieren. Dieser Impfstoff heißt SPf66 (Serum *Plasmodium falciparum* Version 66) und basiert auf chemisch synthetisierten 45-Aminosäure-Peptiden, die aus Fraktionen von 4 verschiedenen Proteinen von *Plasmodium falciparum* stammen [23]. Der Impfstoff wurde in Kolumbien von dem Wissenschaftler Manuel Patarroyo entwickelt, der sein Patent an die Weltgesundheitsorganisation verschenkte. Schnell wurde er in Kolumbien als biomedizinischer Held gefeiert.^6^ Pilotversuche in Afrika zeigten jedoch eine eher geringe Wirkung. Die Zahl der Erstinfektionen mit *P*. *falciparum* wurde um 28 % gesenkt. Bei *P*. *vivax* zeigte SPf66 jedoch keine Wirkung. Das Gleiche galt für Krankenhauseinweisungen wegen schwerer Malaria [24]. Darüber hinaus zeigten Nachuntersuchungen, dass das Auftreten klinischer Malaria bei Kindern, die SPf66 erhielten, statistisch signifikant höher war. Dieser Effekt hängt auch mit der Dosis zusammen. Am ausgeprägtesten war er bei Kindern, die eine hohe Dosis (1,0 mg) von SPf66 erhielten [25, 70]. Heute spielt SPf66 keine Rolle mehr als potenzieller Impfstoff gegen Malaria. Andere Impfplattformen zeigen wesentlich bessere Wirkungen und Immunreaktionen.

### RTS,S/AS01

Eines der besten Ergebnisse wird derzeit mit dem Impfstoff RTS,S/AS01 erzielt. Der Impfstoff, der unter dem Namen Mosquirix™ vermarktet wird, wird von der britischen Fa. GlaxoSmithKline hergestellt. Dieses Unternehmen hatte so hohe Erwartungen an sein Produkt, dass die für die Produktion erforderliche Infrastruktur aufgebaut und fertiggestellt wurde, bevor die ersten Ergebnisse der Wirksamkeitsstudien vorlagen [26]. In Studien erreichte RTS,S/AS01 eine Wirksamkeit von 30 % gegen unkomplizierte Malaria [27]. Ab April 2019 testet die WHO den Impfstoff in Afrika (Malawi, Kenia und Ghana) [27, 28]. Da RTS,S/AS01 der erste Impfstoff ist, der in einer Phase-4-Studie unter realen epidemiologischen Bedingungen getestet wird, stellt sich die Frage, ob die Entwicklung eines wirksamen Malaria-Impfstoffs nun in die Endphase eingetreten ist [27]. Allerdings gibt es auch kritische Punkte. So ist die Wirksamkeit mit 30 % recht gering [29, 71]. Darüber hinaus ist es möglich, dass RTS,S/AS01 die Sterblichkeit bei jungen Mädchen erhöht. Dies wurde in Studien gezeigt, aber es gibt verschiedene Interpretationen. So könnten die Ergebnisse auch statistisch bedingt sein (z. B. durch eine niedrigere Gesamtsterblichkeit in der weiblichen Kontrollgruppe). Weitere Studien und mehr Forschung sind erforderlich, um mehr Informationen zu erhalten [30, 72]. Auch die Dosierung und Auffrischung von RTS,S/AS01 ist derzeit Gegenstand der Forschung. Studien zeigen, dass bei älteren RTS-geimpften Kindern ein signifikanter Anstieg von schwerer Malaria zu verzeichnen ist, wenn sie keine Auffrischung erhalten haben [31, 73]. Die Dosierung ist eine weitere Frage, die durch Forschung geklärt werden muss [32].

### PfSPZ (PfSPZ-CVac)

PfSPZ ist ein weiterer Kandidat, der derzeit untersucht wird. Der Impfstoff wird von Sanaria Inc. in Rockville, Maryland, entwickelt. PfSPZ, das unter dem Namen Sanaria® vermarktet wird, verwendet strahlengeschwächte Sporozoiten, die sich weder teilen noch Krankheiten verursachen können. Diese geschwächten Sporozoiten sollen schützende Immunreaktionen hervorrufen. Der Name PfSPZ leitet sich von Pf für *Plasmodium falciparum* und SPZ für Sporozoiten ab. PfSPZ hat in kleinen Studien bereits gute Ergebnisse gezeigt [33, 34]. Drei Dosen von 5,12 • 10^4^ PfSPZ verhinderten eine Infektion bei 9 von 9 Freiwilligen. Zehn Wochen nach der letzten Dosis wurden die Probanden kontrolliert mit Malaria infiziert [34]. Wie bei anderen Impfungen und Medikamenten ist der Schutz mit PfSPZ dosisabhängig. Mit einer Dosis von 5,12 • 10^4^ PfSPZ wurde ein 100 %iger Schutz erreicht, mit einer Dosis von 3,2 • 10^3^ ein 67 %iger Schutz und mit einer Dosis von 1,28 • 10^4^ ein 33 %iger Schutz [34]. PfSPZ, auch als PfSPZ-CVac bezeichnet, ist ein hochwirksamer Impfstoffkandidat. Als Teil von kombinierten Massenmedikamentenverabreichungs- und Massenimpfungsprogrammen könnte er zur Eliminierung von Malaria eingesetzt werden, zumindest in geografisch definierten Gebieten [34].

### Gentechnisch veränderte Plasmodien in der Impfstoffforschung

Gentechnisch veränderte Plasmodien spielen in der Impfstoffforschung eine wichtige Rolle. Ein Beispiel ist *„upregulated in infective sporozoites gene 3“* (UIS3). Dieses Gen ist essenziell für die Infektion des frühen Leberstadiums. *UIS3*-defiziente Sporozoiten infizieren Hepatozyten, sind aber nicht in der Lage, Infektionen im Blutstadium zu verursachen. Daher führen sie nicht zu Krankheiten. Diese defizienten Sporozoiten eignen sich für eine mögliche Impfung. Die Immunisierung mit *UIS3*-defizienten Sporozoiten bietet einen vollständigen Schutz gegen infektiöse Sporozoiten in einem Nagetiermodell [35]. Das Leberstadium von *Plasmodium*-Infektionen bleibt eines der besten Ziele für abgeschwächte Lebendimpfstoffe [36, 74].

### Testosteron in der Impfstoffforschung

Testosteron könnte in der Impfstoffforschung eine wichtige Rolle spielen. Studien haben gezeigt, dass das Wirtstestosteron einen Einfluss auf Plasmodien hat. So zeigte eine Studie von 1991, dass Testosteron die Selbstheilung von *Plasmodium chabaudi* verringert. Diese Verringerung hängt von der Dauer der Vorbehandlung des Wirts mit Testosteron ab. Bei einer einwöchigen Vorbehandlung war die Selbstheilung auf etwa 60 %, bei 2 Wochen auf 40 % und bei 3 Wochen auf 0 % reduziert [37, 75]. Dieser Verlust der Selbstheilungskräfte korreliert mit einer zunehmenden Expression von 5 Proteinen in den Milzzellen von Mäusen, die 3 Wochen lang mit Testosteron behandelt wurden, und einer gesteigerten Fähigkeit, die durch Concanavalin A ausgelöste proliferative Reaktion von T‑Zellen zu stimulieren [37]. Diese Ergebnisse wurden jedoch nur in vivo und nicht in vitro gezeigt. Daher kann davon ausgegangen werden, dass der Einfluss von Testosteron nicht direkt, sondern indirekt ist, d. h. durch einen Testosteronmetaboliten oder durch testosteroninduzierte Faktoren [37, 38].

## Mögliche Angriffspunkte von mRNA-Impfstoffen gegen Malaria

Zum aktuellen Zeitpunkt kann nicht gesagt werden, wie genau zukünftige mRNA-Impfstoffe gegen Malaria funktionieren werden. Das liegt zum einen daran, dass sich die Impfstoffe noch in der Forschungsphase befinden. Darüber hinaus sind Krankheitserreger wie *Plasmodium* komplexe Einzeller. Die Entwicklung von Medikamenten und Wirkstoffen gegen diese Protisten ist bekanntermaßen schwierig, zeitintensiv und teuer [39, 40]. Auf der anderen Seite hat die mRNA-Impfstofftechnologie mehrere Vorteile. Dazu gehört die hohe Flexibilität, schnell auf z. B. Mutationen zu reagieren [41, 76]. Im Folgenden werden unterschiedliche Angriffspunkte, die für zukünftige Impfstoffe infrage kommen, identifiziert und beschrieben. Da die wissenschaftliche Literatur allerdings aktuell noch spärlich ist, ist dieser Abschnitt des Artikels deutlich spekulativer. Trotzdem ist es nicht unmöglich, mögliche Angriffspunkte zu identifizieren.

Es ist davon auszugehen, dass Oberflächenproteine eine relevante Rolle bei der Entwicklung spielen. Diese spielen eine entscheidende Rolle bei Pathogen-Wirt-Interaktionen, da sie als exponierte Strukturen als Erste mit dem Wirt in Kontakt kommen und z. B. für das Eindringen in die Zelle wichtig sind. Als potenzielle Antigene sind sie auch wichtig für die Immunantwort des Wirts [42, 77]. Das „Spike“-Protein der mRNA-Impfstoffe gegen SARS-CoV‑2 ist ein solches Oberflächenprotein. Gleiches gilt für das „circumsporozoite protein“ (CSP) von *Plasmodium*, das von RTS,S/AS01 angegriffen wird. Oberflächenproteine (und Membranen i. Allg.) spielen auch in der Evolution eine wichtige Rolle [43, 44]. So weisen Plastiden beispielsweise mehrere Membranen auf. Dies kann auf die Endosymbiose während der Evolution zurückgeführt werden. Bemerkenswert ist hier der Apicoplast, ein nichtfotosynthetisches Plastid, das in einigen *Apicomplexa* wie *Plasmodium*-Spezies und *Toxoplasma gondii* vorkommt und wahrscheinlich durch sekundäre Endosymbiose entstanden ist.^7^ Der Apicoplast besitzt 4 Membranen [45]. Er ist ein relevantes Ziel für Medikamente und Impfstoffe [78]. Es ist jedoch fraglich, wie realistisch ein mRNA-Impfstoff ist, der auf den Apicoplast abzielt. Oberflächenproteine sind wahrscheinlich das realistischere Ziel. Heute wissen wir, dass 20–30 % aller exprimierten Proteine in der Zellmembran untergebracht sind. Beim Menschen trägt etwa ein Viertel der vom Genom kodierten Proteine mindestens einen Abschnitt von Sequenzen, die als Transmembrandomäne vorhergesagt werden. Neben der Infektion spielen Oberflächenproteine wichtige Rollen bei der Kommunikation, der Sensorik und der Energieerhaltung [46]; ein breites Spektrum für potenzielle Ziele von Antikörpern und Impfstoffen.

### Circumsporozoite protein

**Figure 4:**
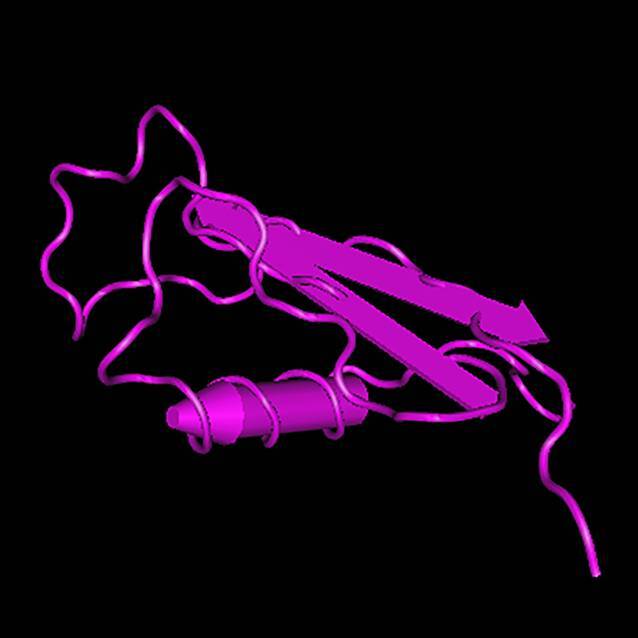

Doch welche Oberflächenproteine könnten für eine mRNA-basierte Impfung geeignet sein? Am wahrscheinlichsten ist wohl eine Impfung, die auf dem CSP basiert. Abb. 4 zeigt die 3D-Struktur des CSP. Die Struktur besteht aus 3 Regionen: dem N‑Terminus, einer Wiederholungsregion mit 4 Aminosäuren und dem C‑Terminus. Der N‑Terminus ist in der Lage, Heparinsulfatproteoglykane zu binden. Der C‑Terminus besitzt eine thrombospondinähnliche „type‑1 repeat (TSR) domain“ [47]. CSP spielt eine wichtige Rolle im Lebenszyklus von *Plasmodium*. Es wird während der Bildung von Sporozoiten in der Stechmücke produziert und spielt eine wichtige Rolle bei der Wanderung des Parasiten vom Stechmückenwirt zum Säugetierwirt und der anschließenden Infektion von Leberzellen [79]. CSP (wie auch seine rekombinante Form) erscheint als ein stark verlängertes Protein (R(h) 4,2 bzw. 4,58 nm). Hochauflösende Mikroskopie zeigt flexible, stäbchenförmige Strukturen mit einem bandartigen Aussehen [47, 80]. Als potenzielles Impfstoffziel hat CSP mehrere Vorteile. Erstens ist CSP hoch konserviert und kommt in verschiedenen Stämmen von Malariaerregern vor (sowohl beim Menschen als auch bei nichtmenschlichen Primaten). Diese Konservierung deutet darauf hin, dass CSP durch natürliche Selektion erhalten geblieben ist. Mutationen in konservierten Sequenzen sind selten. Zweitens ist CSP das Protein, das im ersten zugelassenen Impfstoff gegen Malaria verwendet wird. RTS,S/AS01 ist der erste Impfstoffkandidat, der eine Phase-4-Studie unter realen epidemiologischen und operativen Bedingungen erreicht hat [27]. Diese Punkte machen CSP zu einem guten Kandidaten für zukünftige mRNA-basierte Impfstoffe. *PubMed* listet bereits erste Studien auf, die eine robuste Immunantwort zeigen. So konnten Forscher in Mäusen nach Injektion von CSP-mRNA eine funktionelle und schützende Immunantwort gegen den Erreger *Plasmodium berghei* nachweisen. Verwendet wurde ein transgener Parasitenstamm von *P*. *berghei* (PfCSP steht für „*Plasmodium falciparum* circumsporozoite protein“). Die Ergebnisse machen ihn zu einem überzeugenden Kandidaten für weitere Untersuchungen und deuten darauf hin, dass eine CSP-basierte mRNA-Impfung sehr wahrscheinlich ist [81]. Es ist jedoch fraglich, ob diese Impfung bereits 2022 in die klinische Phase eintreten kann.

### Merozoite surface protein-1

Es gibt weitere Proteine, die für die mRNA-Forschung von Interesse sind. MSP‑1 ist eines von ihnen. MSP steht für Merozoitenoberflächenprotein. MSP spielen eine wichtige Rolle bei der Infektion von roten Blutkörperchen. Sie befinden sich auf der Merozoitenoberfläche und sind mit Glycophosphatidylinositol, einem Glykolipid, an der Membran verankert. MSP‑1 wird zu Beginn der Schizogenese synthetisiert und spielt eine entscheidende Rolle bei der Invasion der roten Blutkörperchen. Der MSP-1-Komplex bindet an Spectrin, ein Oberflächenprotein der roten Blutkörperchen. Eine ähnliche Rolle spielt auch MSP‑2, obwohl seine Funktion noch nicht geklärt ist. Ebenfalls unbekannt sind die Funktionen von MSP‑3, MSP‑6, MSP‑7 und MSP‑9 [48]. MSP‑1 ist für die Impfstoffforschung von besonderem Interesse. Impfstoffe und andere Medikamente, die auf die Merozoiten in ihrem ungeschlechtlichen erythrozytären Stadium abzielen, zielen normalerweise auf MSP‑1 ab. Die komplexe Struktur von MSP stellt jedoch eine Herausforderung für die Entwicklung von Impfstoffen dar. MSP sind nicht so konserviert wie CSP und weisen Sequenzvariationen zwischen verschiedenen Plasmodiumarten auf [49]. Dies stellt eine große Herausforderung für die Entwicklung von Impfstoffen dar und macht die Entwicklung von Impfstoffen zwar nicht unmöglich, wirft aber dennoch die Frage auf, ob Impfstoffe auf der Grundlage anderer Proteine nicht einfacher zu realisieren sind. Die mRNA von MSP‑1 ist 5466 Basenpaare lang [50]. Neben MSP‑1 ist auch MSP‑3 als Impfstoffantigen von Interesse [82].

### *Plasmodium falciparum* reticulocyte-binding homologue 5

Auch Rh5 spielt eine Rolle bei der Invasion von roten Blutkörperchen. Rh5 ist das kleinste Protein der Rh-Familie. Rh steht für Retikulozytenbindungsproteinhomolog. Zu der Familie gehören Rh1, Rh2a, Rh2b, Rh4 und Rh5. Die Besonderheit von Rh5 ist das Fehlen einer Transmembrandomäne. Dies ist einzigartig unter den Rh-Proteinen [51]. Es spielt eine wichtige Rolle bei der Erythrozyteninvasion, da Rh5 mit dem Erythrozytenrezeptor Basigin (BSG) interagiert [52, 83]. Basigin ist ein Glykoprotein, das u. a. im Kohlenhydratstoffwechsel und bei der Immunsignaltransduktion eine Rolle spielt. Darüber hinaus ist BSG eine Determinante des Ok-Blutgruppensystems [53]. Studien haben bereits gezeigt, dass sowohl monoklonale als auch polyklonale Anti-Rh5-Antikörper in der Lage sind, Rh5-BSG-Interaktionen zu blockieren [51, 84]. Rh5 gilt als potenzieller Kandidat für Impfstoffe im Blutstadium. Das macht Rh5 auch für die mRNA-Forschung interessant, aber es gibt auch Schwierigkeiten. Rh5 funktioniert nicht isoliert, sondern nur als Teil eines Multiproteinkomplexes, der aus dem Rh5-interagierenden Protein, dem cysteinreichen Proteinantigen und P113 besteht. Dieser Komplex bindet besser an die Erythrozytenoberfläche als Rh5 allein [51, 54]. Dieser Punkt könnte mit fortschreitender Forschung wichtig werden. Darüber hinaus spielen Polymorphismen als Barrieren eine wichtige Rolle bei der Impfstoffentwicklung, zumindest für Impfstoffe im Blutstadium. Proteine, die während der Invasion dem Immunsystem ausgesetzt sind, weisen oft eine gewisse Diversität auf, die vermutlich auf den Druck des Immunsystems zurückzuführen ist [55]. Ein gutes Beispiel dafür ist AMA1, das bereits als Antigenkandidat für Impfstoffe im Blutstadium getestet wurde. Rh5 scheint konservierter zu sein, was die Entwicklung von Impfstoffen begünstigt [51].

### Pfs25

Pfs25 wird auf der Oberfläche der Zygote und der Ookinetenform von *Plasmodium* exprimiert und ist somit ein Antigen des Sexualstadiums. Antikörper, die gegen Pfs25 wirken, sind in der Lage, die Entwicklung von *P*.-*falciparum*-Oozysten vollständig zu blockieren. Aus diesem Grund ist Pfs25 ein interessanter Kandidat für Impfstoffe, die die Übertragung blockieren [56]. Im Laufe der Jahre sind mehrere Kandidaten und Studien erschienen, die Pfs25 als hochwirksames Antigen beschreiben. Monoklonale Antikörper, die auf Pfs25 abzielen, verhindern nachweislich die Übertragung vom Menschen auf die Stechmücke, indem sie den Übergang vom Ookineten zur Oozyste blockieren [57]. Anders als bei Mäusen konnte die Impfung beim Menschen jedoch keine robusten Antikörpertiter aufbauen. Dies ist wahrscheinlich darauf zurückzuführen, dass menschliche CD4+-T-Zellen Pfs25 (Pfs25-IMX313) schlecht erkennen [58]. Dies ist wichtig für die Entwicklung eines wirksamen mRNA-Impfstoffs gegen Malaria. Wenn man davon ausgeht, dass eine mRNA-Impfung in erster Linie dazu dient, das Individuum zu schützen und nicht die Übertragung zu verhindern, ist diese fehlende Erkennung ein großer Nachteil. Dank der laufenden Forschung sind Impfstoffe auf Pfs25-Basis, die eine robuste Immunreaktion hervorrufen, wahrscheinlich. Um jedoch schnelle und wirksame mRNA-Impfstoffe bereitzustellen, eignen sich andere Proteine wahrscheinlich besser.

## Fazit für die Praxis

mRNA-Impfstoffe befinden sich seit mehreren Jahren in der Entwicklung. Doch erst die aktuelle COVID-19-Pandemie hat praktisch eine medizinische Revolution ausgelöst. In Rekordzeit wurden funktionierende und hochwirksame Impfstoffe gegen SARS-CoV‑2 entwickelt. Es ist daher nicht überraschend, dass die zuständigen Firmen ihren Fokus auf weitere Pathogene ausweiten. In der Zukunft werden wir verschiedene mRNA-Impfstoffe gegen verschiedene Krankheiten haben. Da Malaria eine der gefährlichsten Krankheiten der Welt ist, steht eine mögliche mRNA-Impfung recht weit oben auf den verschiedenen Interessenlisten. Für diese Impfung kommen verschiedene Proteine als Zielantigene infrage. Das Circumsporozoite protein ist wahrscheinlich der beste Kandidat. Aber auch andere Antigene haben das Potenzial, die Grundlage für eine wirksame mRNA-Impfung zu bilden. Diese Überlegungen sind im Moment jedoch noch sehr spekulativ. Die Vergangenheit zeigte, dass die Entwicklung von Malariaimpfstoffen mit einigen Herausforderungen verbunden ist. Mit RTS,S/AS01 befindet sich aktuell lediglich ein Kandidat in weiteren „field trials“. Somit ist es sehr schwer abzuschätzen, wann ein mRNA-Impfstoff gegen Malaria für Studien zur Verfügung stehen wird. Was jedoch mit Sicherheit gesagt werden kann, ist, dass wir einem funktionsfähigen und wirksamen Malaria-Impfstoff immer näherkommen. Die in diesem Text beschriebenen Antigene besitzen ein gutes Potenzial, um in zukünftigen Impfstoffplattformen zum Einsatz zu kommen. Teilweise finden sie jetzt schon Verwendung.

## Footnotes

1 RKI (2015) Malaria: RKI-Ratgeber. Robert Koch-Institut. https://www.rki.de/DE/Content/Infekt/EpidBull/Merkblaetter/Ratgeber_Malaria.html.

2 Spectrum News Staff (2021) BioNTech says it will create an mRNA-based vaccine to prevent malaria. Spectrum News 1. https://spectrumnews1.com/ky/lexington/news/2021/07/26/biontech-malaria-vaccine-mrna-covid.

3 CureVac, Schüler T (2018) CureVac entwickelt innovative mRNA-Impfstoffe gegen Influenza und Malaria. CureVac. https://www.curevac.com/2018/02/18/curevac-entwickelt-innovative-mrna-impfstoffe-gegen-influenza-und-malaria/.

4 BMZ (2021) Neue Partnerschaft für Entwicklung, Frieden und Zukunft: Ein Marshallplan mit Afrika. Bundesministerium für wirtschaftliche Zusammenarbeit und Entwicklung. https://www.bmz.de/de/laender/marshallplan-mit-afrika.

5 Majambere S (2021) Ending malaria in Africa needs to focus on poverty: quick fixes won’t cut it. The Conversation. https://theconversation.com/ending-malaria-in-africa-needs-to-focus-on-poverty-quick-fixes-wont-cut-it-169205.

6 Brown P (1999) Scientist whose dream of beating disease came true. The Guardian. https://www.theguardian.com/uk/1999/jul/24/paulbrown.

7 Der Name *Apicomplexa* leitet sich u. a. von diesem Apicoplast ab.
